# Decomposition Odour Profiling in the Air and Soil Surrounding Vertebrate Carrion

**DOI:** 10.1371/journal.pone.0095107

**Published:** 2014-04-16

**Authors:** Shari L. Forbes, Katelynn A. Perrault

**Affiliations:** Centre for Forensic Science, University of Technology Sydney, Sydney, NSW, Australia; New Mexico State University, United States of America

## Abstract

Chemical profiling of decomposition odour is conducted in the environmental sciences to detect malodourous target sources in air, water or soil. More recently decomposition odour profiling has been employed in the forensic sciences to generate a profile of the volatile organic compounds (VOCs) produced by decomposed remains. The chemical profile of decomposition odour is still being debated with variations in the VOC profile attributed to the sample collection technique, method of chemical analysis, and environment in which decomposition occurred. To date, little consideration has been given to the partitioning of odour between different matrices and the impact this has on developing an accurate VOC profile. The purpose of this research was to investigate the decomposition odour profile surrounding vertebrate carrion to determine how VOCs partition between soil and air. Four pig carcasses (*Sus scrofa domesticus* L.) were placed on a soil surface to decompose naturally and their odour profile monitored over a period of two months. Corresponding control sites were also monitored to determine the VOC profile of the surrounding environment. Samples were collected from the soil below and the air (headspace) above the decomposed remains using sorbent tubes and analysed using gas chromatography-mass spectrometry. A total of 249 compounds were identified but only 58 compounds were common to both air and soil samples. This study has demonstrated that soil and air samples produce distinct subsets of VOCs that contribute to the overall decomposition odour. Sample collection from only one matrix will reduce the likelihood of detecting the complete spectrum of VOCs, which further confounds the issue of determining a complete and accurate decomposition odour profile. Confirmation of this profile will enhance the performance of cadaver-detection dogs that are tasked with detecting decomposition odour in both soil and air to locate victim remains.

## Introduction

The decomposition of organic matter is a complex series of chemical reactions primarily driven by microbial enzymes. These reactions result in the production of volatile organic compounds (VOCs) as by-products that are subsequently released into the surrounding environment [1]–[3]. Their dispersal is ensured by their physicochemical properties, i.e. low molecular weight, high vapour pressure, and low boiling point [4]. Many of these VOCs are attributed to the distinctive odour associated with decomposition and the decay of organic materials. Decomposition odour has been studied extensively in the environmental sciences to address issues of malodourous compounds produced by waste treatment plants [5]–[7], landfills [8], and composting [9]–[11], as well as off-flavours and odour compounds that impact water and food quality [12]–[13]. More recently, decomposition odour has been investigated in the forensic sciences to determine the key compounds used by flies and beetles [2], [14]–[17] and canines [3], [18]–[22] to locate the target source of the odour (i.e. carrion or human remains).

The search for human remains has grown in importance as a result of global catastrophes resulting from natural and man-made disasters. Locating human remains is important for the identification and resolution of a victim's family [23]–[24] but is also important for reducing the threat of pollutants in air, water and soil that may impact public health and safety [25]. The fact that canines can differentiate between odours emitted from living and recently deceased individuals as well as human remains in various stages of decomposition, makes them one of the preferred search techniques for disaster victim recovery [18], [26]–[27]. Cadaver detection dogs (also known as human remains detection dogs) may be tasked with locating cadavers, body parts, soft tissue, decomposition fluid, blood and/or bone [28]. They can detect a scent either by sniffing the air currents to locate the dispersed scent cone from a victim, or by tracking the distinctive volatile compounds adhering to the soil and surrounding vegetation [29].

A consistent VOC profile of decomposition odour is still being investigated and debated by the forensic community [22], [30]. Studies have attempted to chemically profile the decomposition odour in a range of decomposition environments, including remains deposited on the surface [3], [21]–[22], buried [18]–[19], [31], recovered from water [32], and trapped beneath rubble [33]–[34]. The majority of studies involve soil as a deposition environment since this represents a large proportion of the cadaver dog's searches.

Although a considerable number of decomposition VOCs have been reported in the literature, very few compounds consistently appear across decomposition studies. Inconsistencies in the VOC profile of decomposition odour have been attributed to variation in the sample collection and sample analysis methods, the type of remains utilised in the study, and biotic and abiotic factors which are determined by the decomposition environment [2], [22]. For example, Vass et al. [18]–[19] collected decomposition VOCs using a system of pipes placed within a grave and a sampling hood placed above the grave. Both collection techniques were conducted *in situ* using sorbent traps to collect the volatile compounds released from the human remains for subsequent analysis by gas chromatography-mass spectrometry (GC-MS). The study was conducted over several years and the study site was located in the southern region of the USA. In contrast, Brasseur et al. [31] investigated decomposition VOCs in animal grave sites. The method involved collection of soil samples following burial and excavation of porcine remains. VOC collection was carried out *ex situ* in the laboratory. VOCs were trapped on a cartridge containing an adsorbent filter and eluted using solvent desorption for analysis by comprehensive two-dimensional gas chromatography – time-of-flight mass spectrometry (GC×GC-TOFMS). The study was carried out for six months and the study site was located in Belgium. Given the variation in sample collection technique (*in situ* versus *ex situ*), sample analysis (GC-MS versus GC×GC-TOFMS), type of remains investigated (human versus pig), and the location of each study (USA versus Belgium), it is perhaps not surprising that Vass et al. [18]–[19] identified more than 400 specific compounds while Brasseur et al. [31] identified 20–34 compounds specific to decomposition. However, consistent decomposition odour profiles have been reported when similar sample analysis methods are utilised for surface deposition studies, as referenced in the literature [3], [22].

While inconsistencies in the VOC profile of decomposition odour have been attributed to variation in sample collection and analysis techniques, there has been minimal research or discussion on the impact of the sample collection matrix to the overall VOC profile. In order to generate a complete and accurate decomposition odour profile, it is important that samples are collected from both the air and soil surrounding a body as cadaver dogs can use odour detected in both matrices to locate victim remains, particularly when environmental conditions (e.g. wind, temperature) are not conducive to the natural dispersion of VOCs in air. The degree to which decomposition VOCs partition between soil and air is not currently known and sample collection from previous studies has predominantly occurred using only one matrix.

The aim of this study was to compare the VOC profile in the air above and soil below porcine remains deposited on the soil surface over the complete decomposition process to determine whether the sample collection matrix changes the VOC profile detected. Such information is valuable to cadaver detection dog handlers as it demonstrates the proportion of the decomposition VOC profile that is retained in the soil even after dispersion in the air.

## Materials and Methods

### Ethics statement

The study utilised domestic pig (*Sus scrofa domesticus* L.) carcasses weighing approximately 70 kg as analogues for human decomposition. Pig carcasses are considered an acceptable model in decomposition studies due to their physiological and biochemical similarities [35]–[37] and are often used due to ethical, legal and economic restrictions [3]. Carcasses were purchased from Hawkesbury Valley Meat Processors, a licenced abattoir in Sydney, Australia. Animal ethics approval was not required as the experimental subjects were not killed specifically for the purposes of the research and were purchased postmortem. Carcasses were wrapped in two layers of plastic to prevent insects accessing the remains during transport to the site.

The field site was private land belonging to the University of Technology Sydney. No specific permissions were required as the land is zoned for educational and research purposes and the field studies did not involve endangered or protected species. The site is located in open eucalypt woodland in western Sydney, Australia (33°38S, 150°39E). The soils in this area consist of a sandy clay topsoil underlain by shale clays and sandstone bedrock. The topsoil at the study location is acidic and typically ranges between pH 4–5. The dominant tree species are forest red gum (*Eucalyptus tereticornis*), grey box (*Eucalyptus molluccana*), thin-leaved stringybark (*Eucalyptus eugenoides*), and broad-leaved ironbark (*Eucalyptus fibrosa*). The understory shrubs are dominated by the invasive weed lantana (*Lantana camara*), with a few, scattered native species. The ground layer is a mix of native and exotic graminoids and forbs, with large-leaved rush (*Lomandra longifolia*) and kangaroo grass (*Themeda australis*) abundant.

Four carcasses were placed on their side directly on the soil surface with anti-scavenging cages containing small mesh wire (∼1 cm) positioned over the remains. A VOC-Mole™ Soil Probe (Markes International Ltd, UK), adjacent to the abdomen and between the forelegs and hindlegs of each carcass, was probed into the soil to a depth of approximately 30 cm and remained *in situ* for the period of study. Four corresponding control sites without a carcass were established approximately 20 m from the experimental sites and were set up in the same manner to monitor the natural production of VOCs in the surrounding air, soil and vegetation.

The study was carried out during the summer months in Australia from January 15 to March 15, 2013 (a postmortem interval of 59 days). Climatic conditions at the field site were monitored using a Hobo Weather Station equipped with a Hobo U30 No Remote Communication (NRC) data logger (OneTemp, Marleston). The data logger was used to measure ambient temperature (°C), relative humidity (%), solar radiation (W/m^2^), wind speed (m/s), wind direction (ø), and rainfall (mm).

The decomposition stage was characterised by observing the carcasses on each sample collection day, photographing the remains, and documenting the visual postmortem changes to the carcass. Determination of the decomposition stage each sampling day was made by the same researcher to reduce the degree of subjectivity which is inherent in visual observations. Decomposition stages are reported in both experimental days and accumulated degree days (ADD) to facilitate comparison with other decomposition studies. The sample collection regime was determined based on the rate of decomposition. Samples were collected every second day for the first ten days, twice a week for the next three weeks, and every two weeks thereafter.

### VOC sample collection

**Figure 1:**
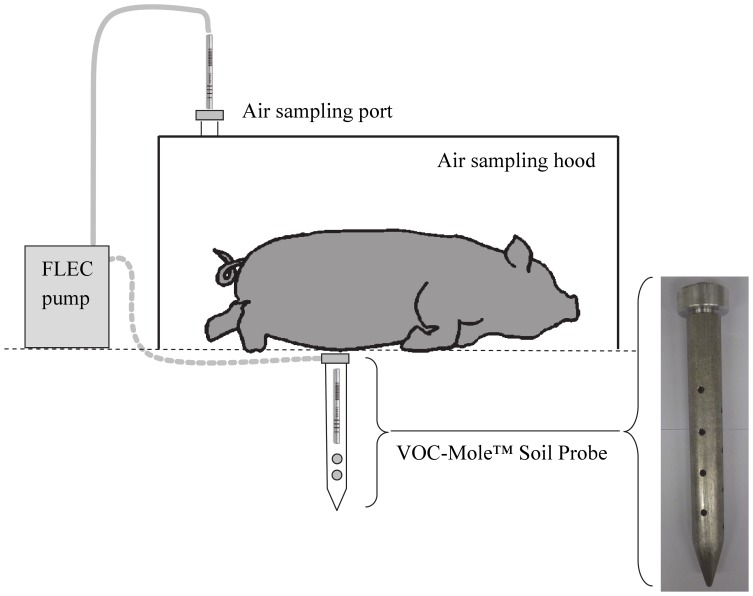
Schematic of VOC sample collection from air and soil surrounding pig carcasses.

A dynamic sampling technique was used to collect the VOCs in the air above, and the soil below, each of the four pig carcasses (Figure 1). Following removal of the anti-scavenging cage and prior to sampling the air above each carcass, a stainless steel hood (dimensions: 130×90×60 cm; volume: 702 L) was placed over the remains to allow the headspace to accumulate for a period of 30 minutes, thus preventing wind or rain dispersal of the VOCs during this time. A Field and Laboratory Emission Cell (FLEC 1001) constant flow air pump (Markes International Ltd, UK) was used to collect the VOCs onto dual-sorbent thermal desorption tubes comprising of Tenax TA and Carbograph 5TD (Markes International Ltd, UK) attached to the sampling port of the hood. A flow rate of 100 mL/min was maintained for 10 minutes, collecting a total volume of 1 L of headspace gases onto the sorbent tubes. The FLEC 1001 pump was also used to collect the VOCs within the VOC-Mole™ Soil Probe onto tubes containing the same dual-sorbent. A flow rate of 100 mL/min was maintained for 30 minutes, collecting a total volume of 3 L of soil headspace gases onto the sorbent tubes. An air and soil sample were collected separately from each of the four carcasses, producing a total of four experimental air samples and four experimental soil samples for each sampling day.

The same experimental design was used to collect samples from the air and soil of the four corresponding control sites, producing a total of four control air samples and four control soil samples for each sampling day. All sample collection tubes were wrapped in aluminium foil and stored in an air-tight, sealed glass jar for transport to and from the field site. Two field blank samples were collected, one prior to the first sample collection and one immediately following the last sample collection on each sampling day.

All sample tubes were injected with an internal standard (10 µL of 20 ng/µL bromobenzene in methanol) following the EPA Method (TO-17) *Determination of Volatile Organic Compounds in Ambient Air Using Active Sampling onto Sorbent Tubes*. Standards were purchased from Sigma-Aldrich and Fluka to represent the common VOCs and classes of compounds reported in the literature for vertebrate decomposition. Standards were used to confirm retention times and mass spectral identifications and included: alkanes (heptane, undecane, 2-methyl pentane); alcohols (ethanol, 1-pentanol, 1-hexanol, 1-hexanol 2-ethyl); aldehydes (hexanal, heptanal, octanal, nonanal); ketones (2-butanone, 2-pentanone, 2-hexanone, 2-nonanone); aromatic hydrocarbons (benzene, ethyl benzene, ρ-xylene, ο-xylene, toluene, styrene, naphthalene); nitrogen-containing compounds (indole, pyridine, putrescine, cadaverine); carboxylic acids (propanoic acid, butanoic acid, hexanoic acid); and sulphides (dimethyl sulphide, dimethyl disulphide).

### Gas Chromatography-Mass Spectrometry (GC-MS) analysis

Sample analysis was carried out utilizing a Unity 2 series thermal desorber (Markes International Ltd, UK) and an Agilent 6890N GC System coupled to an Agilent 5973N Mass Selective Detector (TD-GC-MS).

Each sample underwent a two-step desorption; primary desorption of the sample took place at 300°C for 4 minutes following a leak check and nitrogen dry purge. The sample was re-condensed at −10°C on a general purpose cold trap consisting of Carbopack B (60/80 mesh) and Carboxen 1000-type sorbent (60/80 mesh). The cold trap was then rapidly heated for secondary desorption at 300°C for 3 minutes following another 1 min nitrogen dry purge. Following desorption all tubes were conditioned for 30 minutes at 330°C.

An Agilent DB-VRX capillary column was used (30 m×0.25 mm ID×1.4 µm film thickness). The GC oven started at an initial temperature of 35°C which was held for 4 minutes, then increased to 80°C at a rate of 3°C/min, then further increased to 120°C at a rate of 10°C/min before ramping to the final temperature of 220°C at a rate of 40°C/min which was held for 7 minutes. The MS was operated in full electron ionization (EI) scan mode with a mass range of 40–450 m/z.

### Data analysis

To generate a list of compounds, library search and area reports were produced using Agilent Chemstation. The library search report was generated using the National Institute of Standards and Technology (NIST) Mass Spectral library. A minimum match threshold of 70 was selected. Compounds were removed during pre-processing if they were below this threshold, present at comparable levels in field blank samples, or if they resulted from sorbent or column bleed. A comparison of control and experimental samples produced a list containing compounds of interest that were found exclusively in experimental samples, or where the level of the compound was above control and field blank levels. Internal standard normalisation was performed on the peak area of each compound of interest. The resulting normalised peak area represented a relative amount of each compound of interest present on the sorbent tube and is not representative of the absolute concentration being emitted into the surrounds by the carcasses. The aim of this approach was to establish trends within the air and soil independently and to determine whether these trends were similar between groups. The two matrices were sampled using analytically independent collection techniques and a direct comparison was therefore not feasible.

Principal component analysis (PCA) was used to visualize the structure of the data for air and soil independently. This statistical analysis is particularly useful where dimensionality of the data is high and often where possibility for replication is low. This typically represents situations where variability and noise in the data set are high, eradicating the possibility of using traditional hypothesis testing techniques to determine significant differences in the data. Normalised peak areas were summed by compound class for each stage of decomposition and PCA was performed using The Unscrambler X version 10.3 (CAMO Software). The resulting biplots reduce the dimensionality of the data by showing a visual representation of the variables (i.e. loadings) that are responsible for discriminating each of the stages of decomposition. In this manner, important compound classes could be identified that discriminated the stages of decomposition.

## Results

### Stages of decomposition

Decomposition stages were adapted from [35] and [38] but varied in the later stages of decomposition. Carcasses were characterised as being in the fresh stage from day 0 to day 1 (ADD 24.40). All carcasses were in full bloat on day 2 (ADD 48.16) and showed early signs of active decay by day 4 (ADD 106.95). The active decay stage continued until day 10 (ADD 244.28) when early signs of advanced decay and partial mummification became evident. On day 14 (ADD 340.85), all carcasses were characterised as being in the advanced decay stage with extensive mummification. From day 17 (ADD 413.73) onwards, the carcasses were skeletonized in the head, limbs, and ribs but with large amounts of mummified tissue remaining until the end of the study on day 59 (ADD 1315.46).

### Climatic conditions

**Figure 2:**
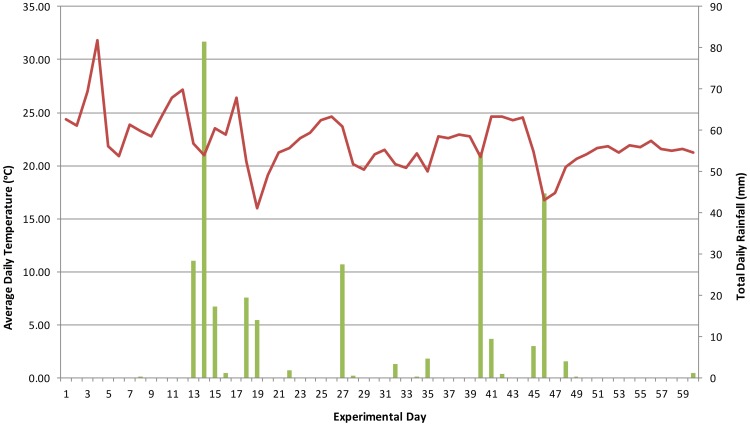
Average temperature and rainfall data for the study site (January - March, 2013).

The average temperature throughout the period of study was 22.3°C with an average maximum of 31.8°C and an average minimum of 16.0°C (Figure 2). The temperature demonstrated some extreme highs such as day 3 reaching a maximum of 47.2°C, and lows such as day 19 reaching a minimum of 13°C but generally fluctuated within the averages. A total rainfall of 324 mm was recorded but occurred sporadically throughout the study (Figure 2). Periods of high rainfall coincided with the transition from the early advanced decay stage to the skeletonization stage making the remains appear wet even though there was little moisture remaining in the soft tissue. The site experienced an average humidity of 81% during the summer months. Average wind speed was negligible (0.009 m/s). The average solar radiation, i.e. the rate at which diffuse solar energy falls on a unit horizontal surface area per second, was 93.8 Watts per square metre (W/m^2^).

### VOC profile of air above the remains

The normalised peak area ratios were calculated from the internal standard and grouped by major compound classes. Table 1 demonstrates the major VOCs detected in the air samples based on the stages of decomposition (with the exception of the fresh stage as VOCs were not detected above background levels during this stage). Major VOCs were determined as those compounds that appeared in more than two experimental replicates on each sampling day and were detected consistently for a particular stage of decomposition. The sulphides were the most prevalent chemical class with dimethyl disulphide (DMDS) and dimethyl trisulphide (DMTS) appearing throughout all stages of decomposition. Nitrogen-containing compounds including trimethylamine, methenamine and 2,6-dimethylpyrazine were detected during the active decay stage while methenamine and benzonitrile appeared during skeletonization. Indole was the only aromatic detected throughout all decomposition stages reported, however 2-pentylfuran regularly appeared throughout the active decay, advanced decay and skeletonization stages. Carboxylic acids also appeared frequently during these stages and included acetic acid, propanoic acid (including 2-methyl-), butanoic acid (including 2-methyl- and 3-methyl-), and pentanoic acid. Ketones were not detected until the active decay stage with 2-butanone, 2-pentanone, and 2-piperidinone appearing frequently thereafter. Phenol was the most prevalent alcohol during the active and advanced decay stages and other alcohols were detected sporadically throughout the decomposition process. Hydrocarbons (mostly C_5_-C_20_ straight chain alkanes) were only detected during the skeletonization stage. Esters, aldehydes and other compounds were only detected occasionally throughout the trial.

**Table 1:** Major VOCs consistently identified in experimental air samples throughout the decomposition stages (excluding fresh).

	Bloat	Active Decay	Advanced Decay	Skeletonization	Literature Citation
*Sulphide*					
Sulphur dioxide	▴	▴			[1], [18], [19], [21], [32]
Dimethyl disulphide	▴	▴	▴	▴	[14], [18], [19], [20], [21], [26], [31], [32], [43]
Dimethyl trisulphide	▴	▴	▴	▴	[14], [18], [19], [21], [26], [31], [32], [41]
*Nitrogen-containing*					
Trimethylamine		▴			[21], [22], [27], [34], [40]
Methenamine		▴		▴	[18], [19], [30]
2,6-dimethylpyrazine		▴			[22]
Benzonitrile				▴	[18], [21], [22]
*Aromatic*					
Benzene				▴	[19], [27], [30], [32]
Indole	▴	▴	▴	▴	[20], [21], [27], [40]
2-pentylfuran		▴	▴	▴	[20], [27], [40]
*Carboxylic Acid*					
Acetic acid		▴	▴	▴	[22], [34], [41]
Propanoic acid			▴	▴	[20], [22], [27], [40]
2-methylpropanoic acid		▴	▴	▴	[41]
Butanoic acid		▴		▴	[3], [20], [21], [22], [40]
2-methylbutanoic acid		▴	▴	▴	[3], [21], [22], [27]
3-methylbutanoic acid		▴	▴	▴	[3], [21], [22], [27], [34]
Pentanoic acid		▴	▴		[20], [21], [22], [40]
2-methylhexanoic acid		▴			[27]
Benzoic acid		▴			[21]
*Ester*					
Methyl acetate				▴	NR*
*Aldehyde*					
3-methylbutanal		▴	▴	▴	[22], [26], [30], [34]
*Ketone*					
2-butanone		▴		▴	[22], [26], [30], [32], [34]
2-pentanone		▴	▴	▴	[22], [32], [34]
2-piperidinone		▴	▴		[21]
1-phenylethanone				▴	[21], [31], [34]
*Alcohol*					
1-propanol	▴				[3], [22]
2-propanol			▴	▴	[26]
3-methyl-1-butanol			▴	▴	[21], [22]
Phenol		▴	▴		[3], [18], [21], [22], [26], [31], [34], [41]
4-methylphenol	▴	▴	▴		[3], [21], [22], [26]
4-(1,1-dimethylpropyl)phenol		▴			NR
2-phenylethanol				▴	[21]
*Hydrocarbon*					
Pentane				▴	[30]
Hexane				▴	[19], [26], [27], [30], [32], [34]
Heptane				▴	[18], [26], [30], [32], [34]
Octane				▴	[22], [26], [30], [32]
Tridecane				▴	[34], [41]
Pentadecane			▴	▴	[34], [41]
1-pentadecene				▴	NR
Eicosane				▴	[21]

*NR =  Not Reported in Literature.

**Figure 3:**
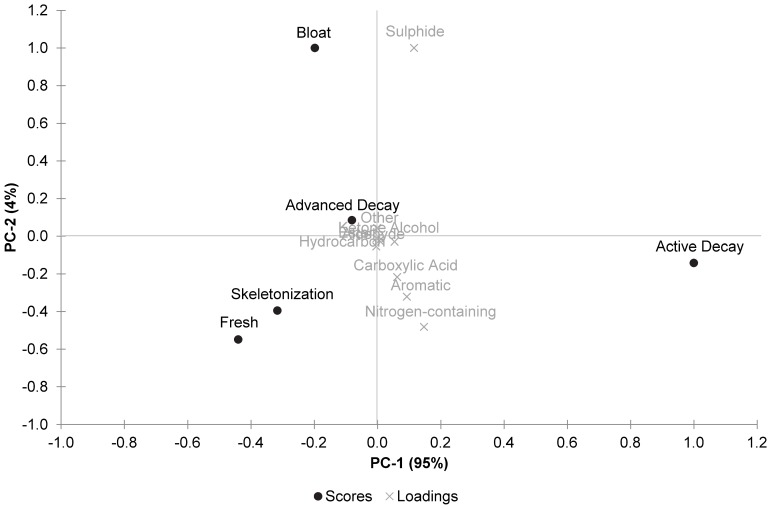
PCA biplot of the calculated scores and loadings for the air experimental samples.

In order to characterize the relationship of the VOCs between decomposition stages, PCA was conducted to preserve the variation in the original data set while allowing for visualisation of data groupings. A biplot of the principal component scores and loadings for the air samples is shown in Figure 3. Each stage is clearly distinct with the fresh and skeletonization stages appearing the most similar in terms of VOC profile, likely due to reduced levels of VOC production during these stages. The bloat stage was characterised by high levels of sulphides and the active decay stage was characterised by a predominance of sulphides, aromatics, carboxylic acids, and nitrogen-containing compounds. The advanced decay stage (including extensive mummification) was mostly characterised by sulphides with other compound classes having a minor influence. The results correlated with the PCA biplots generated on each individual experimental day (data not shown) which confirmed that the experimental samples from day 2 (bloat) to day 14 (advanced decay) were clearly separated from the control samples but thereafter became difficult to distinguish. The control and experimental samples could not be distinguished on day 0 (fresh), which is to be expected given the minimal decomposition and lack of VOCs produced that day. The major compound classes responsible for the variation were the sulphides, aromatics, carboxylic acids, alcohols, and nitrogen-containing compounds. The other classes were not discriminating in the air samples.

### VOC profile of soil below the remains

The VOCs detected in the soil samples demonstrated a more dynamic profile than the air samples. The normalised peak area ratios were calculated from the internal standard and grouped by major compound classes. Table 2 demonstrates the major VOCs detected in the soil below the remains based on the stages of decomposition (with the exception of the fresh stage as VOCs were not detected above background levels during this stage). Sulphides were prevalent in the soil samples with a larger number of sulphur-containing compounds detected in soil compared to air. Sulphides were detected every sampling day except day 0 and included DMDS, DMTS, with dimethyl sulphide only detected during the skeletonization stage. The nitrogen-containing compounds, specifically trimethylamine and 3-methylpyridine, were detected during active decay and skeletonization whereas benzonitrile and benzylnitrile were only detected during skeletonization. Aromatic compounds were not detected until the active decay stage but then appeared frequently throughout the process of decomposition. Although carboxylic acids were not as prevalent in soil samples, the corresponding esters of the carboxylic acids detected in air samples were predominant in soils during active decay. Other notable differences between the soil and air samples were the large number of alcohols, ketones and hydrocarbons detected consistently in soil from the active decay stage onwards. Aldehydes also appeared regularly during the active decay, advanced decay, and skeletonization stages.

**Table 2:** Major VOCs consistently identified in experimental soil samples throughout the decomposition stages (excluding fresh).

	Bloat	Active Decay	Advanced Decay	Skeletonization	Literature Citation
*Sulphide*					
Dimethyl sulphide				▴	[14], [18], [19], [20], [21], [26], [32]
Dimethyl disulphide	▴	▴	▴	▴	[14], [18], [19], [20], [21], [26], [31], [32], [43]
Dimethyl trisulphide	▴	▴	▴	▴	[14], [18], [19], [21], [26], [31], [32], [41]
2,4-dithiapentane		▴			[14], [34]
3-methylthiophene		▴			[21]
*Nitrogen-containing*					
Trimethylamine		▴		▴	[21], [22], [27], [34], [40]
3-methylpyridine		▴		▴	[21]
Benzonitrile				▴	[18], [21], [22]
Benzylnitrile				▴	[21]
*Aromatic*					
Indole		▴	▴	▴	[20], [21], [27], [40]
2-ethylfuran		▴		▴	[32]
2-butylfuran				▴	NR*
2-pentylfuran		▴	▴	▴	[20], [27], [40]
2-methyltetrahydrofuran			▴	▴	NR
2-butyltetrahydrofuran				▴	NR
Styrene		▴		▴	[18], [19], [26], [30], [41]
*Carboxylic Acid*					
Acetic acid		▴			[22], [34], [41]
2-methylbutanoic acid		▴			[3], [21], [22], [27]
Benzoic acid				▴	[21]
*Ester*					
Methyl acetate				▴	NR
Ethyl acetate		▴			NR
Propyl acetate		▴			[21], [32]
Propanoic acid, 2-methyl-,ethyl ester		▴			[32]
Propanoic acid, ethyl ester		▴			NR
Butanoic acid, 1-methyl-, ethyl ester		▴			NR
Butanoic acid, ethyl ester		▴			[20], [32]
Butanoic acid, 2-methyl-, ethyl ester		▴			NR
Butanoic acid, 3-methyl-, butyl ester		▴			[21]
Butanoic acid, methyl ester		▴			NR
Butanoic acid, propyl ester		▴			NR
Butanoic acid, butyl ester		▴			[20], [21]
Pentanoic acid, ethyl ester		▴			NR
*Aldehyde*					
Hexanal		▴	▴		[19], [20], [27], [30], [32], [40]
Octanal				▴	[3], [19], [20], [22], [30]
Nonanal		▴	▴	▴	[18], [19], [20], [27], [30], [41]
3-methylbutanal		▴	▴	▴	[22], [26], [30], [34]
2-phenylpropenal		▴			NR
2-methyl-prop-2-enal		▴			NR
*Ketone*					
2-butanone		▴	▴	▴	[22], [26], [30], [32], [34]
3-methyl-2-butanone				▴	[22], [34]
2-pentanone		▴	▴	▴	[22], [32], [34]
2-hexanone		▴		▴	[32]
2-heptanone		▴		▴	[3], [20], [22], [27], [32]
2-octanone				▴	[3], [22]
3-octanone			▴	▴	[3], [22]
2-nonanone		▴		▴	[3], [19], [22], [27], [32]
2-undecanone		▴		▴	[3], [22], [27]
2-nonadecanone		▴		▴	NR
1-phenylethanone		▴		▴	[3], [20], [31], [34]
3-methyl-3-buten-2-one		▴			NR
1,7,7-trimethylbicyclo[2.2.1]heptan-2-one				▴	NR
*Alcohol*					
Ethanol		▴	▴	▴	[19], [21], [26], [30], [32]
1-propanol		▴	▴	▴	[3], [22]
2-propanol			▴	▴	[26]
2-methyl-1-propanol		▴	▴	▴	[21], [32], [34]
2-propen-1-ol		▴			NR
1-butanol		▴	▴	▴	[21], [22], [32], [34]
2-butanol		▴		▴	[21], [22], [34]
2-methyl-1-butanol		▴	▴	▴	NR
3-methyl-1-butanol		▴	▴	▴	[21], [22]
1-pentanol		▴	▴	▴	[20], [21], [22], [27], [32], [34], [40]
2-pentanol		▴		▴	[32], [34]
2-hexanol				▴	NR
2-heptanol				▴	NR
1-octanol				▴	[21], [22]
1-nonanol				▴	[41]
Phenol		▴	▴	▴	[3], [18], [21], [22], [26], [31], [34], [41]
4-methylphenol		▴		▴	[3], [21], [22], [26]
2-phenylethanol		▴		▴	[21]
*Hydrocarbon*					
Pentane		▴		▴	[30]
Hexane		▴	▴	▴	[19],
1-hexene				▴	[32], [34]
Heptane		▴	▴	▴	[18], [26], [30], [32], [34]
1-heptene				▴	[32], [34]
Octane		▴	▴	▴	[22], [26], [30], [32]
1-octene			▴	▴	[22]
Tridecane				▴	[34], [41]
Pentadecane				▴	[34], [41]
1-pentadecene				▴	NR
8-heptadecene				▴	NR
α-pinene		▴	▴	▴	[32], [34]
β-pinene				▴	NR
1-methyl-2-pentylcyclopropane			▴		NR
1-methyl-4-(1-methylethyl)-1,4-cyclohexadiene				▴	NR

*NR =  Not Reported in Literature.

**Figure 4:**
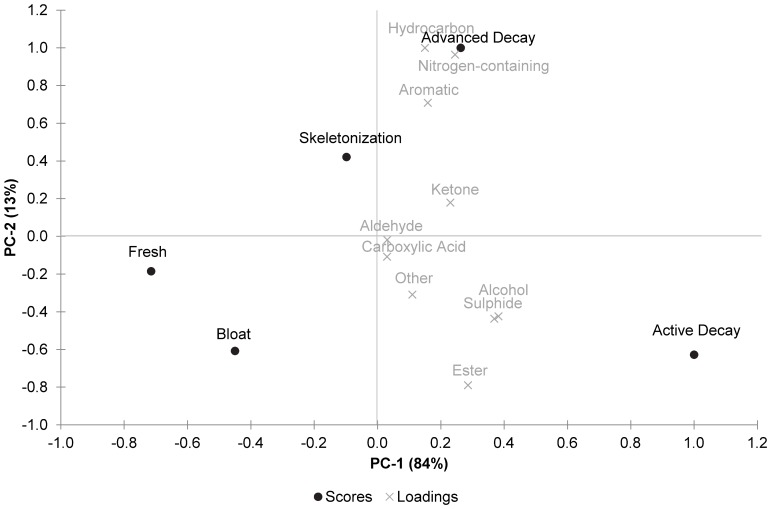
PCA biplot of the calculated scores and loadings for the soil experimental samples.

A biplot of the principal component scores and loading for the soil samples is shown in Figure 4. The decomposition stages were clearly separated with fresh and bloat appearing most similar in terms of VOC profile. The active decay stage was characterised by high levels of sulphides, alcohols, and esters although all compound classes were detected during this stage. The advanced decay stage was characterised by high levels of hydrocarbons and high levels of indole, the latter being classed as both an aromatic and nitrogen-containing compound in the data analysis. The results correlated with the PCA biplot generated on each individual experimental day (data not shown) which confirmed that the experimental samples from day 2 (bloat) to day 48 (skeletonization) were clearly separated from the control samples. The major compound classes responsible for the separation were the sulphides, alcohols, esters, ketones, aromatics, hydrocarbons and nitrogen-containing compounds. Carboxylic acids and aldehydes were not discriminating in the soil samples.

### Comparison of trends in air and soil VOC profiles

**Figure 5:**
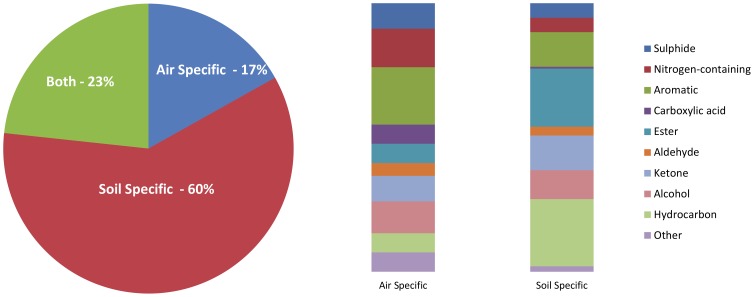
Percentage of total compounds specific to air and soil including dominant classes.

Sample collection from air and soil surrounding the carrion provided complementary data. Although a large number of VOCs were detected in both the air and soil samples, the majority of compounds could be traced to environmental sources and were not specific to the decomposition process. A total of 249 VOCs of interest were detected using both techniques with 42 compounds (17%) specific to the air samples and 149 compounds (60%) specific to the soil samples. The remaining 58 compounds were common to both air and soil but represent only 23% of the total number of compounds of interest (Figure 5). As shown by the proportional bar graphs, the majority of air-specific VOCs were represented by sulphides, nitrogen-containing compounds, aromatics, and alcohols as these typically appeared throughout all stages of decomposition. In contrast, the soil samples were dominated by aromatics, esters, ketones, alcohols and hydrocarbons which were only detected during the active decay, advanced decay, and skeletonization stages of decomposition.

A comparison of the PCA biplots (see Figures 3 and 4) confirm these differences in loadings for the compound classes and also show variation in the scores for decomposition stages. Experimental and control samples collected from the air above the remains could be clearly separated for days 2–14 but were not distinguishable for the fresh and skeletonization stages. This finding suggests that the VOC profile of the air rapidly returns to baseline (control) levels by the final stage of decomposition, at which point it is comparable to the VOC profile for the fresh stage. However, experimental and control samples collected from the soil could be clearly separated for days 2–48 suggesting that the VOC profile, even during the final stage of decomposition, is still detectable above baseline (control) levels and is distinctly different to the fresh stage. This prolonged period of detection may be a result of the increased number of VOCs detected in soil (207 compounds) compared to in air (100 compounds). The increased number of VOCs in soil could be related to the soil microbial community or to the adsorption of VOCs to soil particles causing longer retention.

## Discussion

### VOC profiles and stage of decomposition

The chemical processes of soft tissue degradation that release the characteristic VOCs associated with decomposition odour have been reported extensively in the literature [1], [3], [14], [18]–[22], [26]–[28], [30]–[34], [39]–[41]. The current study demonstrated comparable VOC profiles to many previous studies in terms of the dominant VOCs detected in both animal and human remains (see Tables 1 and 2); including dimethyl sulphide, DMDS, DMTS, trimethylamine, indole, phenol, hexane, heptane, octane, and a range of straight chain- and methylated- aldehydes, ketones, alcohols and carboxylic acids. This finding demonstrates that key VOCs are consistently being produced in both air and soil throughout the decomposition process, even in distinctly different geographical locations such as Australia, Belgium [3], [21], [31], Greece [26], [32]–[34], USA [1], [18]–[20], [27]–[28], [30] and Canada [22]. Given the variation in climate, geology, and ecology (including soil microbial species) across these locations, it is valuable to identify these consistencies across studies as they may assist in determining the core VOC profile of decomposition odour.

The majority of decomposition VOCs identified in the air above the remains were released during the bloat, active decay, and advanced decay stages, with reduced levels (but not a reduced number) of compounds detected during skeletonization. These stages are often attributed with the pungent odour of decomposition detectable by humans as well as the VOCs detected by cadaver-detection dogs, vertebrate and invertebrate scavengers. It was during these stages of decomposition that the identified VOCs were most prevalent in terms of number of compound classes and relative amount of each major class i.e. sulphides, aromatics, carboxylic acids, and alcohols.

Although decomposition odour was detected in the air during the bloat stage, it was not as readily detectable in soil at the equivalent time. The majority of decomposition VOCs identified in the soil below the remains were detected during the active decay, advanced decay, and skeletonization stages. The active decay stage coincided with the liquefaction of soft tissue and the release of the body's constituents into the surrounding soil forming a cadaver decomposition island [40]. Thereafter, the soil microbial community could utilise these nutrients producing additional VOCs to those produced by the remains, thus allowing for their detection in soil throughout the later stages of decomposition.

### Factors affecting VOC detection in air and soil

The types of VOCs detected in air versus soil did not directly correlate with their molecular weights and volatility. Overall, there were fewer compounds detected in the air samples above the remains compared to the soil samples below the remains. This may have resulted from the rapid dispersion of VOCs in air due to wind, evaporation, or other environmental factors. It could have also resulted from the physical, chemical and microbiological properties of the soil. VOCs are active in both gas and liquid phases and as a result can move through the network of soil pores by vapour diffusion [4]. Volatile compounds can sorb to soil particles however adsorption and desorption is dependent on the polarity of the compound, the soil texture (including pore space), the organic/mineral content, and the soil-water content [43]. In dry soil, water will compete with VOCs for adsorption sites on the soil surface whereas in wet soil, the water will act as a solvent for polar VOC molecules.

Natural volatile emissions from soil are typically low suggesting that soil acts as a volatile sink [44]. This theory is supported by the reduced number of VOCs detected in control soils in the present study (data not shown). When a nutrient source is added to a soil, however, the soil microbial community can rapidly metabolise these nutrients, producing volatile products which are utilised by other organisms in the microbial food chain or released into the atmosphere as excess products [4]. Bacteria and fungi have a significant capacity to decompose, mineralise, and accumulate organic matter, in the process producing large quantities of highly diverse volatiles. The presence of decomposed remains will act as a nutrient source when placed in a soil environment [42], thus increasing the number of VOCs being produced and detected in the soil.

Many of the VOCs detected in the experimental soil samples could be attributed to the decomposition process based on previous literature, however several classes of compounds, specifically hydrocarbons, esters, alcohols, and ketones, were detected in higher levels and more consistently in soil when compared to the air samples. The VOCs emitted by certain bacteria and fungi present inside and on a vertebrate have been reported by [39] and the VOCs emitted by certain bacterial and fungal genera in soil have been reported by [4]. The dominant classes of compounds emitted by bacteria include alcohols, alkanes, alkenes, and ketones followed by esters, pyrazines, lactones, and sulphides. The dominant classes of VOCs emitted by fungi include alcohols, ketones, terpenes, alkanes and alkenes. Based on the *in situ* sample collection technique used in this study, it was not possible to differentiate VOCs produced by the decomposition process of vertebrate soft tissue and those produced by the soil microbial community. Hence, specific volatile compounds identified in the soil experimental samples that may be attributed to either the decomposition process [39] or the soil microbial community [4] are detailed herein: indole produced by *Pseudomonas* spp. in soil, and *Bacteroides, Lactobacillus* spp., and *Clostridium* spp. during decomposition; DMDS and DMTS produced by a range of bacteria and fungi in soil and during decomposition (e.g. *Lactobacillus* spp., *Clostridium* spp., *Citrobacter* spp., *Pseudomonas* spp. and *Flavobacterium* spp.); 4-methylphenol produced by *Pseudomonas aeruginosa* and *Staphylococcus aureus* in soil, and *Clostridium* spp. and *Fusobacterium* spp. during decomposition; 3-methyl-1-butanol produced by *Escherichia coli, Pseudomonas fluorescens* and *Streptomyces* spp. in soil, and *Staphylococcus* spp. during decomposition; 2-phenylethanol produced by *Escherichia coli, Lactobacillus* spp. and many other bacteria in soil, and *Pseudomonas* spp. and *Clostridium* spp. during decomposition; and short chain ketones produced by *Penicillium* spp. in soil, and *Pseudomonas* spp., *Acinetobacter* spp., *and Bacillus* spp. during decomposition. Pyrazine is also prominent in bacterial emissions; 2,6-dimethylpyrazine and 3-methylpyridine (one fewer nitrogens) were detected in the air and soil samples, respectively.

VOCs are organic compounds that have a high vapour pressure and are therefore readily vaporised at room temperature. For this reason, temperature must also be considered when comparing the proportion of VOCs in air and soil in an outdoor environment. Day-time temperatures during the summer months of the trial typically exceeded 22–24°C which may have impacted the number and types of VOCs released into the surrounding environment. Temperature will also impact the microbial species composition in vertebrate carrion and soil communities. The volatile profile produced by microorganisms growing at cool temperatures will be different to the volatile profile produced by microorganisms growing at ambient temperature [39]. For example, Rajamaki et al. (as reported in [39]) detected higher levels of DMDS – which results from the bacterial degradation of methionine – in the headspace of chicken carcasses as the temperature increased. This finding highlights the potential for trace VOCs that are not detectable in cool temperatures to be subsequently detected as the ambient temperature increases. In the current study, VOC sample collection from air and soil was carried out at the same time each day to minimize variation in ambient temperature other than the natural daily fluctuations. With the exception of Day 19 and 46, the average ambient temperature (which included the cooler night temperatures) rarely dropped below 20°C.

### Advantages of in situ sample collection technique

The higher number of VOCs detected in soil may also be the result of the sample collection technique employed. The use of a VOC-Mole™ Soil Probe offers several advantages to previous soil sampling methods that have utilised syringe extraction of headspace gases [30] or solvent extraction from sorbents [31]. The soil sampling technique was able to detect the same compounds reported by [31] with a considerably shorter collection time (30 mins) and without the need for solvent extraction since the VOCs are thermally desorbed directly to the GC inlet. The soil probes were also able to collect a larger number of decomposition VOCs (207 compounds) when compared to the methods used by Vass [30] (50 compounds) and Brassuer et al. [31] (20–34 compounds). This is most likely attributed to the *in situ* collection method, which reduces the loss of volatile compounds during collection, transport, and subsequent analysis in the laboratory. It may also result from the permanent nature of the apparatus in the soil, thereby allowing VOCs to continually partition between the soil and the probe, and providing a better representation of soil VOCs at the time of sample collection. The probe also demonstrated enhanced sensitivity as several compounds reported as being human specific in grave soils (e.g. pentane, undecane, and higher levels of 3-methylbutanal) [30] were able to be detected at low concentrations in the experimental soils, confirming that they are also associated with animal remains.

## Conclusions

This study has demonstrated that the collection of VOCs in the air above decomposed remains and the soil below decomposed remains provides complementary information about the number and type of VOCs resulting from the decomposition process. Although more VOCs were detected in soil, this may be attributed to the production of additional VOCs by the soil microbial community and/or the sorption of these compounds to soil surfaces allowing for their detection over longer periods of time (i.e. throughout the later stages of decomposition). It could also have resulted from the novel *in situ* sample collection technique employed for sampling soil VOCs. Additionally, environmental factors may account for the reduced number of VOCs detected in the air.

The results of this study demonstrate that both air and soil sampling techniques are required to identify the broad spectrum of VOCs produced throughout the decomposition process. The use of only one of these techniques will limit the number of VOCs identified and will further confound the detection of the complete decomposition VOC profile. Confirmation of this profile is important for advancing the understanding of decomposition odour and the mechanisms by which cadaver-detection dogs recognise decomposition VOCs when searching for and locating victim remains in soil environments.
